# AGE RELATED CHANGES IN THE MICROBIOME AND THEIR IMPACT ON DISEASE

**DOI:** 10.1093/geroni/igae098.1593

**Published:** 2024-12-31

**Authors:** Louise McCullough

**Affiliations:** McGovern Medical School at UTHealth, Houston, Texas, United States

## Abstract

Trillions of microbes live symbiotically in the host, specifically in mucosal tissues such as the gut. Recent advances in metagenomics and metabolomics have revealed that the gut microbiota plays a critical role in the regulation of host immunity and metabolism, communicating through bidirectional interactions in the microbiota-gut-brain axis (MGBA). The gut microbiota regulates both gut and systemic immunity and contributes to the neurodevelopment and behaviors of the host. Disruption in the balance of gut microbial communities (often referred to as “dysbiosis”) has many detrimental effects in the host including gut and brain inflammation. Reversing these changes may have therapeutic potential. With aging, the composition of the microbiota changes, and emerging studies have linked these shifts in microbial populations to age-related neurological diseases (NDs). Preclinical studies have demonstrated that gut microbiota-targeted therapies can improve behavioral outcomes in the host by modulating microbial, metabolomic, and immunological profiles. We will summarize the role of gut microbiota and microbial metabolites across the lifespan and in disease. We will highlight recent studies investigating 1) microbial changes with aging; 2) how aging of the maternal microbiome can affect offspring health; and 3) the contribution of the microbiome and its metabolites to outcomes after experimental ischemic stroke.

